# Death by a Thousand Ants: Predation on Grasshoppers by Invasive Ants in a Grassland

**DOI:** 10.1002/ece3.72700

**Published:** 2025-12-16

**Authors:** Ryan W. Reihart, Chelse M. Prather

**Affiliations:** ^1^ Department of Biology University of Dayton Dayton Ohio USA

**Keywords:** invasion, orthoptera, prairie food web, stable isotope, tawny crazy ants, tethering

## Abstract

Invasive ants are among the most ecologically disruptive taxa worldwide, often exerting high predation pressure that drives declines in native ant and arthropod diversity. Although their impacts on litter‐dwelling assemblages are well documented, effects on large, mobile aboveground insects remain poorly understood. Here, we report predation by the invasive tawny crazy ant (*Nylanderia fulva*) on adult grasshoppers in a coastal tallgrass prairie in Texas. In tethering experiments, nearly half of grasshoppers (48.7%) were killed and consumed by *N. fulva* within 24 h, demonstrating their collective ability to subdue large herbivores. Complementary stable isotope analyses placed *N. fulva* at an intermediate trophic position (mean = 2.30), consistent with an omnivorous diet dominated by plant‐ and honeydew‐derived resources but supplemented opportunistically by animal prey. These findings extend the known ecological impacts of *N. fulva* , suggesting that predation on grasshoppers may further alter plant–herbivore interactions and threaten the conservation of imperiled tallgrass prairies. Our results highlight the importance of considering both litter and aboveground arthropod communities when assessing the ecological consequences of invasive ants.

## Graphical abstract

Invasive ants are known to disrupt litter‐dwelling arthropod communities, but their impacts on large, mobile aboveground insects are less clear. We document predation by the invasive tawny crazy ant (Nylanderia fulva) on adult grasshoppers in a coastal tallgrass prairie, where nearly half of tethered individuals were killed within 24 h, and stable isotope analyses confirmed an omnivorous diet supplemented by animal prey. These findings suggest that *N. fulva* may alter grassland food webs by reducing grasshopper abundance and richness, with potential consequences for plant–herbivore interactions and prairie conservation.

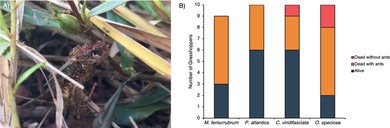

## 1 Introduction

In 2016, as a starting PhD student, one of us (Reihart) stepped foot in a coastal tallgrass prairie for the first time and immediately understood why invasive species can have such profound effects on the composition of communities and function of ecosystems. Within seconds, his feet were covered by hundreds, if not thousands, of ants that also blanketed the ground. Invasive ants often have the advantages of numbers, which is a key to their success in reducing the abundance and species richness of co‐occurring native ant species and non‐ant arthropods (Holway et al. 2002). Although the diet of invasive ants has been implicated as an important mechanism driving their numerical dominance, it remains difficult to not only understand the composition of the diets of invasive ants but also predict the consequences of invasion on native communities (Lach and Hooper‐Bui 2010).

**FIGURE 1:**
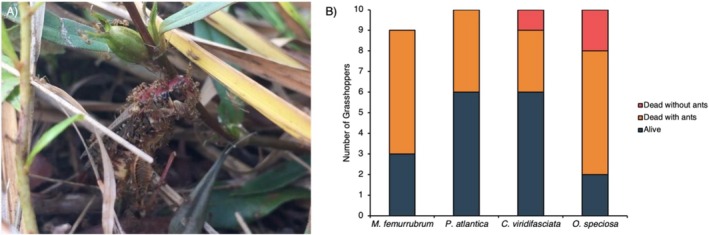
(A) Still image of *N. fulva* swarming a dead, tethered *Melanoplus femurrubrum* during the tethering experiment on July 7, 2016, at 9:30 AM CDT in a coastal tallgrass prairie. Photo Credit: Chelse Prather. (B) Number of grasshoppers found alive (blue), dead with *N. fulva* covering the body (orange), and dead with no *N. fulva* near the grasshopper (red) at the end of the 24‐h tethering experiment. One *M. femurrubrum* lost both legs while tethered and was removed prior to the end of the experiment. Typically, a few grasshoppers out of hundreds become prey after multiple days of tethering experiments, but 48.72% of grasshoppers were found dead within the first 4 h in this experiment.

Originating from South America, invasive populations of *Nylanderia fulva* (Mayr) (tawny crazy ants) in the southeast US exhibit traits that are common among some of the most widespread and destructive invasive ants (Holway et al. 2002). In the US, *N. fulva* forms a large, disjointed supercolony, spanning from Texas to Florida, that lacks genetic differentiation and aggressive conspecific behavior among nests (Eyer et al. 2018). Like other invasive ants, *N. fulva* is a highly opportunistic and omnivorous scavenger that forages on energy‐rich resources, like plant nectaries and aphid honeydew, as well as small‐living and dead arthropod prey (Sharma et al. 2013; Jones 2019; Kjeldgaard et al. 2022). These resources fuel invading *N. fulva* populations that can reach numerical dominance within a year of establishment and drive declines in the diversity of native ants and small, non‐ant litter arthropods (LeBrun et al. 2013). Our understanding of the effects of invasive ants on insect communities may be incomplete, however, because most studies only focus on the effects within ground‐ or litter‐dwelling arthropod assemblages (Holway et al. 2002; Lach and Hooper‐Bui 2010). Here, we report the observation of grasshopper predation by *N. fulva* in the green food web and also conduct stable isotope analysis to determine its trophic position in a coastal tallgrass prairie near the Gulf Coast of Texas (Figure 1A and Video S1).

## 2 Observation

Although at the Texas Institute for Coastal Research and Education at the University of Houston's Coastal Center (UHCC; 29°23′26.96″ N; 95°1′51.95″ W) in July 2016, we were testing the importance of grasshopper diversity on grassland ecosystem function (Prather, Pelini, and Pennings 2017; Laws et al. 2018) and observed *N. fulva* feeding on multiple grasshoppers in these enclosure experiments. We were also finding greater numbers of dead grasshoppers covered in ants throughout the summer compared to other years, and this prompted a few questions: (1) Do these invasive prey upon large, mobile aboveground insects like grasshoppers; and (2) How does the diet of this ant differ relative to other trophic levels in this coastal tallgrass prairie?

## 3 Methods, Results, and Discussion

### 3.1 Tethering Experiment: Does *N. fulva* Prey Upon Grasshoppers?

Our study site at the UHCC contains approximately 121.4 ha of coastal tallgrass prairie, which is dominated by graminoids, forbs, and shrubs (Prather et al. 2018; Reihart et al. 2021). Annual air temperatures at UHCC average 20.90°C, whereas rainfall averages 1070 mm (Prather et al. 2018; Reihart et al. 2021).

Following our above observation, we conducted a tethering experiment to determine if *N. fulva* has the potential to prey upon grasshoppers. We tethered 40 grasshoppers—10 individuals each of two grass feeding species ( *Chortophaga viridifasciata* and *Orphulella speciosa* ) and two mixed feeding species ( *Melanoplus femurrubrum* and *Paroxya atlantica)—with* 50 cm of fishing line and used a modified “leash” that was carefully tightened under the metazonal disk of the pronotum (Belovsky et al. 1990). In our initial observations, it seemed as though ants were feeding upon grasshoppers with a mixed diet. To confirm this, we chose grasshoppers with different diets to determine if the ants selectively preyed upon species with certain diets. The free end of the fishing line was secured to a nail that was inserted into the ground, spaced at least 3 m apart. Usually, in these experiments, a few individuals out of hundreds will be preyed upon over the course of a couple of days (Belovsky et al. 1990; Belovsky and Slade 1993), but high predation rates by other species of invasive ants have been shown on live, ground‐dwelling baits in other systems (Sharp et al. 2025). Grasshoppers that were tethered climbed and jumped on vegetation, such as *Rhynchospora caduca* , *Andropogon gerardii* , *Helianthus* angustafolius, *Myrica cerifera* , and *Rubus argutus* , as normal and fly short distances but could not fly away. Although we did not collect behavioral data on tethered grasshoppers and controls, anecdotally, we did not notice that tethered grasshoppers spent more time on the ground than non‐tethered grasshoppers. Additionally, *N. fulva* workers are often seen climbing on vegetation where grasshoppers spend most of their time. Grasshoppers were tethered around 3 m away from one another; this close spatial distribution means that ants around this area may have been from the same nest. Thus, bait stations were not truly independent from one another, and the results were interpreted accordingly.

Surprisingly, as we were setting up the experiment around 9:30 AM, we observed a single *N. fulva* worker crawl up the hind leg of a red‐legged grasshopper ( *M. femurrubrum* ), bite the grasshopper between the femur and tibia, and curl its gaster so that it was pointing at its opponent—likely using its caustic, formic acid as a chemical weapon (LeBrun et al. 2014). This behavior resulted in the *M. femurrubrum* individual kicking with its hind leg and falling to the ground, at which point, hundreds of *N. fulva* began to swarm the moribund grasshopper (Figure 1A). Within the first 4 h, 48.72% (10 grass feeders and 9 mixed feeders) were dead and covered by *N. fulva* . Of the remaining grasshoppers, 7.69% (2 grass feeders and 1 mixed feeder) were found dead but had no ants on them, whereas 43.59% (9 grass feeders and 8 mixed feeders) survived and were released after 24 h (Figure 1B). For all individuals that were dead besides the one individual attack described above, we did not observe direct attacks, so it is possible the ants were scavenging. However, no other reasons for mortality were observed. We did not observe ants climbing on the tethers to the grasshoppers; they climbed directly onto the grasshoppers' bodies.

These data demonstrate a potential profound collective foraging ability of *N. fulva* to subdue adult grasshoppers under field normal conditions, which affirmatively answers our first question of whether these ants can potentially prey upon large, mobile herbivores. Interestingly, LeBrun et al. (2013) reported that *N. fulva* reduces the abundance and richness of litter‐dwelling herbivores more so than other trophic groups, likely due to predation of eggs and early instars and because herbivores need to be stationary to feed. One caveat from our tethering results to consider is that the limited mobility of grasshoppers may have overestimated predation rates found in the field. We also recognize that these results were likely from just one or a few localized *N. fulva* nests and that further testing is needed to confirm this. Despite this, our tethering results suggest that this preference for herbivores may also hold true for larger, aboveground taxa like grasshoppers, but no comparison to other groups was made here. On the basis of these data, we predict that *N. fulva* has the potential to alter green food webs.

### 3.2 Stable Isotope Analysis: How Does the Diet of *N. fulva* Differ Relative to Other Trophic Levels?

Given that *N. fulva* may prefer herbivores and has the potential ability to subdue adult grasshoppers under field conditions, we used stable isotopes to determine the relative trophic position and relative importance of other insects in the diet of *N. fulva* . We hand‐collected 4 dominant species of C_4_ plants ( *Tripsacum dactyloides* ; *Andropogon gerardii* ; *Schizachyrium scoparium* ; *Rhynchospora caduca* ) and 4 dominant species of C_3_ plants ( *Liatris pycnostachya* ; *Centella erecta* ; *Rubus argutus* ; *Myrica cerifera* ) as baseline species for estimating trophic position. To collect arthropods, we used a sweep net to collect 4 dominant herbivores (grass feeders: *Chortophaga viridifasciata* ; *Orphulella speciosa* ; mixed feeders: *Melanoplus femurrubrum* ; *Paroxya atlantica* ), 2 known omnivores (katydids: *Orchelimum vulgare* ; *Neoconocephalus robustus* ), and 1 known predator (spider: *Rabidosa rabida* ). Because *Nylanderia fulva* is supercolonial in its invaded range with no distinct nest boundaries (Eyer et al. 2018), colonies were characterized by dense aggregations of workers that contained brood and queens and had to be at least 25 m away from other nests that were sampled during this study. At each nest, we hand collected approximately 15–30 workers with a bulb aspirator. All plants and insects collected were immediately put on ice until frozen at −20°C.

Before sending the samples for analysis, samples were dried to a constant mass for 48 h at 60°C, ground to a fine powder, and encapsulated in tin. For non‐ant taxa and plants, each replicate consisted of 1 individual arthropod or 1 leaf from collected plants, except *Myrica cerifera* , which consisted of 3 leaves to meet minimum weight requirements for analyses. Because the petiole and gaster contain residual food particles that can skew the isotopic signature (Tillberg et al. 2006), we removed the petiole and gaster before homogenizing workers. Each replicate *N. fulva* sample consisted of 9–16 homogenized workers to meet minimum weight requirements. Stable isotope analyses were performed at the University of California Davis Stable Isotope Facility using a PDZ Europa 20–20 isotope ratio mass spectrometer (Seron Ltd., Cheshire, UK).

Delta $\left(\delta \right)$ values of plants and insects were calculated using the equation:

$$\delta =\left(\left(\frac{R_{\text{Sample}}}{R_{\text{Standard}}}\right)-1\right)\times 1000$$

where δ is reported in per mil notation (‰), which is representative of the ratio of heavy to light isotopes within a sample ($R_{\text{sample}}$) relative to the ratio of an international standard ($R_{\text{standard}}$; air for nitrogen, Vienna Pee Dee Belemnite for carbon).

Relative trophic position (TP) for *N. fulva* was calculated using the equation:

$$\mathrm{TP}=\lambda +\frac{\left(\delta^{15}N_{N.\;\text{fulva}}-\delta^{15}N_{\text{plants}}\right)\;}{∆N}$$

where λ was equal to the trophic level of the basal food source (e.g., autotroph = 1), $\delta^{15}N_{N.\;\text{fulva}}$ values were directly measured from *N. fulva* , whereas $\delta^{15}N_{\text{plants}}$ was calculated by averaging the δ ^15^N values from vegetation across UHCC. Since plants have a lower tissue turnover rate compared to consumers in terrestrial systems, we used plants from our study site as a baseline for trophic position estimates (Shurin et al. 2005). We used a ∆N (i.e., trophic discrimination factor) of 3.4‰ for *N. fulva* , representative of the standard enrichment per trophic level (Kelly 2000; Post 2002), because we were interested in determining the variation in the trophic position of *N. fulva* , rather than directly interpreting the absolute trophic position in context of the food web at this field site (Kjeldgarrd et al. 2021). Without stable isotope mixing models, we infer relative trophic position rather than quantitative diet percentages of *N. fulva* .

**FIGURE 2:**
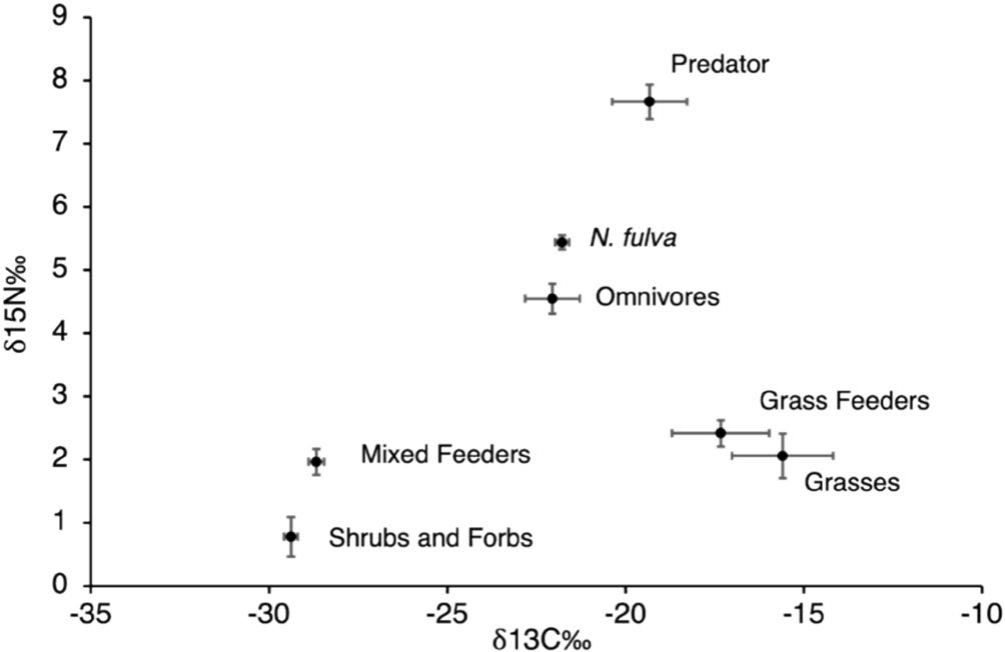
Isotope composition (δ ^13^C and δ ^15^N) of plants, herbivores, omnivores (katydids), a predator ( *Rabidosa rabida* ), and *Nylanderia fulva*. Each group is represented by its mean and SE bars.

We found that *N. fulva* workers varied from 4.49‰ to 6.14‰ in δ ^15^N (Figure 2; mean ± standard error; 5.44 ± 0.11) and −23.07‰ to −19.89‰ in δ ^13^C (Figure 2; −21.78 ± 0.19). To test for differences between δ ^15^N values of *N. fulva* with those of plants, herbivores, omnivores, and predators, we used a Kruskal–Wallis nonparametric test and determined pairwise differences between each trophic group using a Dunn‐Bonferroni test as a post hoc using the package FSA v0.8.25 (Ogle et al. 2023) in Program R version 4.2.2 (R Core Team 2022). When *N. fulva* δ ^15^N values were compared to plants, herbivores, omnivores, and predators, *N. fulva* values were significantly higher than plants (*Z* = 7.09, *p* < 0.001) and herbivores (*Z* = 4.81, *p* < 0.001), but not significantly different from omnivores (*Z* = 1.27, *p* > 0.05) and predators (*Z* = −0.93, *p* > 0.05; Figure 2, Table 1 and Appendix S1). Although *N. fulva* δ ^15^N values were indistinguishable from predators, like other populations of *N. fulva* in Texas (Kjeldgaard et al. 2022), relative trophic position only varied from 2.03 to 2.52 (2.30 ± 0.14) across 20 nests at our site. The lack of variation in the trophic position may indicate that colonies also obtain a large proportion of its *N* from mutualist‐provided honeydew or nectar, which can have a stabilizing effect on δ ^15^N values (Wilder et al. 2011). These results suggest that both animal‐ and plant‐derived resources play an important role in the diet of *N. fulva* , and highlights that dietary flexibility may play a major role in the success of this invasive ant (Kjeldgaard et al. 2022). However, ants are driven by the nutritional demands of the colony, and large prey items may provide colonies with potentially limiting nutrients that may be lacking in plant and hemipteran exudates. As highly omnivorous scavengers, *N. fulva* may be opportunistically preying upon insects and scavenging prey items for micronutrients. At our field site in 2017, *N. fulva* made up approximately 97% of the litter community, and their abundance was limited by the availability of calcium (Reihart et al. 2021). The availability of Ca may be hard to obtain for *N. fulva* for two main reasons: (1) Ca is hypothesized to be scarce in hemipteran honeydew (Reihart et al. 2021); and (2) many small litter arthropods (i.e., oribatid mites, beetles) have a hard exoskeleton that is not easily digestible, where much of the Ca is locked from predators, and may prevent attack from *N. fulva* . Conversely, grasshoppers, which have fleshy abdomens, may represent a Ca‐rich prey source because chewing herbivores often have relatively high tissue concentrations of Ca compared to other groups (Schowalter et al. 1981).

**TABLE 1:** Summary of Z‐scores, *p*‐values, and Bonferroni adjusted *p*‐values from Dunn's Test to determine pairwise differences in δ ^15^N between trophic groups (plants, herbivores, omnivores, *N. fulva* , and predators).

Trophic groups	Z‐score	*p*	Adjusted *p*
*N. fulva* – Plants	**7.09**	**< 0.001**	**< 0.001**
*N. fulva*—Herbivores	**4.81**	**< 0.001**	**< 0.001**
*N. fulva* – Omnivores	1.27	0.20	1.00
*N. fulva* —Predators	−0.93	**0.04**	1.00
Herbivores – Plants	1.71	0.08	0.86
Herbivores – Omnivores	−2.80	**0.01**	0.51
Herbivores – Predators	**−3.91**	**< 0.001**	**< 0.001**
Omnivores – Plants	**4.39**	**< 0.001**	**< 0.001**
Omnivores – Predators	−1.75	0.8	0.80
Plants – Predators	**−4.99**	**< 0.001**	**< 0.001**

*Note:* *p*‐Values are adjusted with the Bonferroni method and were a result of contrasts between trophic groups. Bolded values are statistically significant.

Grasshopper composition and diversity are essential to maintaining the proper functioning of grasslands because critical combinations of species can have strong effects on plant biomass and composition (Laws et al. 2018), soil enzymatic functioning (Prather, Pelini, and Pennings 2017), and microbial biomass and nutrient cycling (Lucas et al. 2021). Mixed feeding orthopterans (e.g., *M. femurrubrum* ), for example, are important for reducing the percent cover of native shrubs (Laws et al. 2018), like wax myrtle ( *Myrica cerifera* ), which has been shown to facilitate the spread of the invasive Chinese tallow ( *Triadica sebifera* ) by providing woody perches for seed‐dispersing birds at our field site (Prather, Strickland, et al. 2017). If *N. fulva* does reduce the abundance and richness of large, aboveground herbivores as we predict on the basis of these data, invading populations of this ant may be a serious threat to the conservation of imperiled coastal tallgrass prairies and other grasslands in the southeastern United States.

## 4 Concluding Remarks

Our observations and results highlight some complexities in the diet of invasive ants but also use this information to make inferences on the potential effects of *N. fulva* on communities and ecosystems. This study suggests that *N. fulva* is a highly opportunistic omnivore that has the potential to reduce the abundance and richness of orthopteran communities in grasslands, adding to a growing list of taxa that invasive ants can impact (Holway et al. 2002; Lach and Hooper‐Bui 2010). Sampling both litter and aboveground insect communities in newly invaded and adjacent uninvaded sites over time may help to better understand how these invasive ants are affecting nymph and adult grasshoppers, as well as other aboveground arthropods. Moreover, isotopic Bayesian mixing models (e.g., MixSIAR) or AA‐CSIA (i.e., Amino Acid‐Compound Specific Isotope Analysis) may be a useful next step to quantify the relative proportion of animal and plant resources in the diet of *N. fulva* . Increasing our understanding of the diets of invasive ants will allow for better predictions of their consequences and potentially help to mitigate some of the long‐term effects that invasions can have on native diversity.

## Author Contributions

**Ryan W. Reihart:** conceptualization (equal), data curation (equal), formal analysis (equal), investigation (equal), methodology (equal), writing – original draft (equal). **Chelse M. Prather:** conceptualization (equal), methodology (equal), resources (equal), supervision (equal), writing – review and editing (equal).

## Conflicts of Interest

The authors declare no conflicts of interest.

## Supporting information


**Appendix S1:** ece372700‐sup‐0001‐Supinfo.zip.

## Data Availability

Associated data (including raw isotope data and grasshopper mortality data) are available from Figshare (Reihart 2024a): https://doi.org/10.6084/m9.figshare.25274230.v1; and (Reihart 2024b): https://doi.org/10.6084/m9.figshare.25276534.v1.
