# Supplementary material for: Death by a Thousand Ants: Predation on Grasshoppers by Invasive Ants in a Grassland
Source: Ecol Evol. 2025 Dec 16;15(12):e72700. doi: 10.1002/ece3.72700 (PMC12706636; doi:10.1002/ece3.72700)
Supplement: Supplementary file 1 — Appendix S1: ece372700‐sup‐0001‐Supinfo.zip. [file ECE3-15-e72700-s001.zip › ECE3_72700__author.docx]

**Ecology**

**Appendix S1. Supplemental Methods**

**Supporting information for:**

Death by a thousand ants: Predation on grasshoppers by invasive ants in a grassland

**Authors:** Ryan W. Reihart and Chelse M. Prather

*Study Site*

We worked in remnant and restored coastal tallgrass prairie at the Texas Institute for Coastal Research and Education at the University of Houston’s Coastal Center (UHCC; 29°23’26.96” N; 95°1’51.95” W). UHCC contains approximately 121.4 ha of coastal tallgrass prairie, which is dominated by graminoids, forbs, and shrubs (Prather et al. 2018; Reihart et al. 2021). Annual air temperatures at UHCC average 20.90°C, while rainfall averages 1,070 mm (Prather et al. 2018; Reihart et al. 2021).

*Stable Isotope Field and Laboratory Methods*

We hand collected 4 dominant species of C_4_ plants (*Tripsacum dactyloides*; *Andropogon gerardii*; *Schizachyrium scoparium*; *Rhynchospora caduca*) and 4 dominant species of C_3_ plants (*Liatris pycnostachya*; *Centella erecta*; *Rubus argutus*; *Myrica cerifera*). To collect arthropods, we used a sweep net to collect 4 dominant herbivores (grass feeders: *Chortophaga viridifasciata*; *Orphulella speciosa*; mixed feeders: *Melanoplus femurrubrum*; *Paroxya atlantica*), 2 known omnivores (katydids: *Orchelimum vulgare*; *Neoconocephalus robustus*), and 1 known predator (spider: *Rabidosa rabida*). Because *Nylanderia fulva* is supercolonial in its invaded range (Eyer et al. 2018), colonies were characterized by dense aggregations of workers that contained brood and queens and had to be at least 25 m away from other nests that were sampled during this study. At each nest, we hand collected approximately 15-30 workers with a bulb aspirator. All plants and insects collected were immediately put-on ice until frozen at -20 ° C.

Before sending the samples for analysis, samples were dried to a constant mass for 48 hours at 60°C, ground to a fine power, and encapsulated in tin. For non-ant taxa and plants, each replicate consisted of 1 induvial arthropod or 1 leaf from collected plants, expect *Myrica cerifera* which consisted of 3 leaves to meet minimum weight requirements for analyses. Because the petiole and gaster contain residual food particles that can skew the isotopic signature (Tillberg et al. 2006), we removed the petiole and gaster before homogenizing workers. Each replicate *N. fulva* sample consisted of 9-16 homogenized workers to meet minimum weight requirements. Stable isotope analyses were performed at the University of California Davis Stable Isotope Facility using a PDZ Europa 20-20 isotope ratio mass spectrometer (Seron Ltd., Cheshire, UK).

*Stable isotope delta* ($\delta$) *values calculation*

*N. fulva* delta $(\delta)$ values were calculated using the equation:

$$\delta=\left( \left( \frac{R_{Sample}}{R_{Standard}} \right)-1 \right) \text{X }1000$$

where $\delta$ is reported in per mil notation (‰), which is representative of the ratio of heavy to light isotopes within a sample ($R_{sample}$) relative to the ratio of an international standard ($R_{standard}$).

*Stable Isotope Trophic Position (TP) Calculation*

Trophic position (TP) for *N. fulva* was calculated using the equation:

$$TP= \lambda+ \frac{(\delta^{15}N_{\mathrm{insect}}- \delta^{15}N_{\mathrm{base}})}{\Delta N}$$

where $\lambda$ was equal to the trophic level of the basal food source (e.g., autotroph = 1), $\delta^{15}N_{\mathrm{insect}}$ values were directly measured from the insects of study, while $\delta^{15}N_{\mathrm{base}}$ was calculated by averaging the $\delta$^15^N values from vegetation across UHCC. $\Delta N$ is representative of the standard enrichment per trophic level of 3.4‰ (Kelly 2000; Post 2002).

**Table S1.** Summary of Z-scores and *P*-values from Dunn’s Test to determine pairwise differences in $\delta$15N between trophic groups (Plants, Herbivores, Omnivores, *N. fulva*, and Predators). *P*-values are adjusted with the Bonferroni method and were a result of contrasts between trophic groups.

| **Trophic Groups** | **Z - score** | ***p-value*** |
| --- | --- | --- |
| *N. fulva ­­*– Plants | **7.09** | **<0.001** |
| *N. fulva* - Herbivores | **4.81** | **<0.001** |
| *N. fulva* – Omnivores | 1.27 | 1.00 |
| *N. fulva* - Predators | -0.93 | 1.00 |
| Herbivores – Plants | 1.71 | 0.86 |
| Herbivores – Omnivores | -2.80 | 0.51 |
| Herbivores – Predators | **-3.91** | **<0.001** |
| Omnivores – Plants | **4.39** | **<0.001** |
| Omnivores – Predators | -1.75 | 0.80 |
| Plants – Predators | **-4.99** | **<0.001** |

**Supplemental Literature Cited**

Eyer, P., B McDowell, L. N. L. Johnson, L. A. Calcaterra, M. B. Fernandez, D. Shoemaker, R. T. Puckett, and E. L. Vargo. 2018. Supercolonial structure of invasive populations of the tawny crazy ant *Nylanderia fulva* in the US. BMC Evolutionary Biology 18:209.

Kelly, J. F. 2000. Stable isotopes of carbon and nitrogen in the study of avian and mammalian trophic ecology. Canadian Journal of Zoology 78:1-27.

Prather, C. M., A. N. Laws, J. F. Cuellar, R. W. Reihart, K. M. Gawkins, and S. C. Pennings. 2018. Seeking salt: herbivorous prairie insects can be co-limited by macronutrients and sodium. Ecology Letters 21:1467-1476.

Post, D. M. 2002. Using stable isotopes to estimate trophic position: models, methods, and assumptions. Ecology 83:703-718.

Reihart, R. W., K. P. Angelos, K. M. Gawkins, S. E. Hurst, D. C. Montelongo, A. N. Laws, S. C. Pennings, and C. M. Prather. 2021. Crazy ants craving calcium: macronutrients and micronutrients can limit and stress an invaded grassland brown food web. Ecology 102:e03263.

Tillberg, C. V., D. P. McCarthy, A. G. Dolezal, and A. V. Saurez. 2006. Measuring the trophic ecology of ants using stable isotopes. Insectes Sociaux 53:65-69.
