# Supplementary material for: Death by a Thousand Ants: Predation on Grasshoppers by Invasive Ants in a Grassland
Source: Ecol Evol. 2025 Dec 16;15(12):e72700. doi: 10.1002/ece3.72700 (PMC12706636; doi:10.1002/ece3.72700)
Supplement: Supplementary file 1 — Appendix S1: ece372700‐sup‐0001‐Supinfo.zip. [file ECE3-15-e72700-s001.zip › ece372700-sup-0002-Supinfo2@Tethering_Video_S1_Metadata.docx]

**Ecology**

**Metadata for Video S1**

**Supporting information for:**

Death by a thousand ants: Predation on grasshoppers by invasive ants in a grassland

**Authors:** Ryan W. Reihart and Chelse M. Prather

**Metadata for Video S1**

Video of *N. fulva* swarming a dead, tethered *Melanoplus femurrubrum* during the tethering experiment on July 7, 2016, at 9:30 AM CDT in a coastal tallgrass prairie.

Video Credit: Chelse Prather
